# Electrical Cardioversion for Wide Complex Tachycardia

**DOI:** 10.7759/cureus.5174

**Published:** 2019-07-19

**Authors:** Abhishek Roka, Tej G Stead, Sidhartha R Ramlatchan, Jose A Rubero, Latha Ganti

**Affiliations:** 1 Emergency Medicine, University of Central Florida College of Medicine, Orlando, USA; 2 Emergency Medicine, Brown University, Providence, USA; 3 Emergency Medicine, Osceola Regional Medical Center, Ellicott City, USA; 4 Emergency Medicine, Envision Physician Services, Orlando, USA

**Keywords:** cardioversion, electrical cardioversion, tachycardia, wide complex tachycardia

## Abstract

We present a case of electrical cardioversion used to treat a hemodynamically unstable wide complex tachycardia (WCT). The patient returned to normal sinus rhythm after being cardioverted with 100 joules (J) on the first attempt. He was admitted to the hospital for cardiac evaluation and ultimately discharged home on flecainide and nebivolol after a negative cardiac workup.

## Introduction

Cardioversion is a process used to restore an irregular heartbeat to its normal rhythm [1]. This can be done through electric shock (electrical cardioversion) or through drugs (pharmacologic cardioversion). Electrical cardioversion requires the use of a defibrillator, to deliver electricity to a patient’s heart. Electrodes are connected to the machine and are placed on the chest of the patient [2-3]. This can be done either via adhesive patches placed on the anterior and either the lateral or posterior chest wall, or via physical paddles. The advantage of paddles is that additional physical force can be applied by the operator to deliver a more effective shock. The electric shock delivered by the defibrillator temporarily stops all electrical activity in the heart, allowing the organ to regain a normal rhythm moments later. The electricity can be delivered in either a monophasic or biphasic manner. Biphasic energy is preferable to monophasic because it can defibrillate more effectively at lower energy levels [4].

Electrical cardioversion can be either synchronized with the peak of the QRS complex (the highest point of the R-wave) or unsynchronized. Synchronized electrical cardioversion is used to treat hemodynamically unstable ventricular and supraventricular rhythms in patients who have a pulse, while unsynchronized cardioversion (defibrillation) is used to treat ventricular tachycardia or ventricular fibrillation without a pulse [5]. Hemodynamic instability is defined as having any of the following signs or symptoms: hypotension, altered mental status, acute congestive heart failure, chest pain, dyspnea, diaphoresis, and/or myocardial ischemia [3].

## Case presentation

A 55-year-old male with a past medical history of paroxysmal atrial fibrillation, hypertension, hyperlipidemia, and gastritis, presented to our emergency department via ambulance with a chief complaint of lightheadedness, associated with nausea and vomiting. The patient reported feeling lightheaded two hours prior to arrival, along with nausea and three episodes of non-bloody emesis. The patient underwent cardiac ablation one month prior for his paroxysmal atrial fibrillation. He denied chest pain, shortness of breath, abdominal pain, syncope, weakness, numbness, or tingling. He received 324 mg of aspirin en route. On physical exam, his heart rate was significantly elevated at 191 beats per minute, and he was hypotensive with a blood pressure of 61/57 mmHg. His pulse oximetry was 98% on room air, and he was afebrile. 

**Figure 1:**
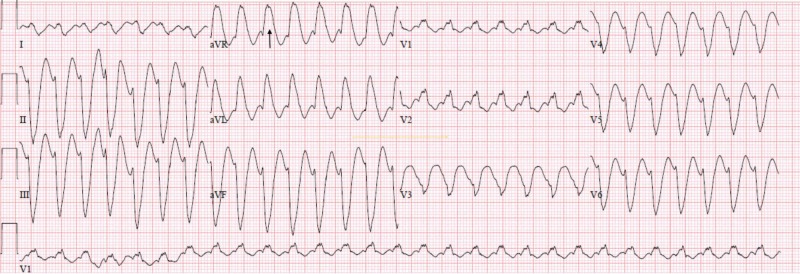
Wide complex tachycardia prior to cardioversion; arrow shows wide QRS complex

The patient's electrocardiogram (EKG) showed wide complex tachycardia (WCT) (Figure 1). Since the patient was hypotensive, and therefore hemodynamically unstable, the decision was made to cardiovert the patient with informed consent. The patient was successfully cardioverted with 100 joules (J) of electricity on a biphasic device.

**Figure 2:**
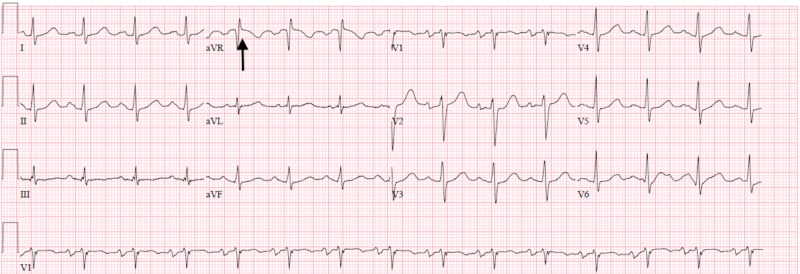
Normal sinus rhythm after cardioversion; arrow shows narrow QRS complex

The patient’s heart rate improved to 89 beats per minute following the cardioversion, and his blood pressure normalized (Figure 2). The patient was no longer lightheaded and nauseated. The patient’s troponin came back at 0.01 ng/mL (normal), he did not have any electrolyte abnormalities or anemia. His thyroid-stimulating hormone level was also normal.

The patient was admitted for cardiac workup, and started on flecainide and nebivolol (a beta1 selective blocker) by the cardiologist then discharged home. The patient remained in normal sinus rhythm throughout his hospital stay.

## Discussion

Our patient presented with a WCT. Eighty percent of WCTs are due to ventricular tachycardia (VT) [5]. The second most common cause is supraventricular tachycardia (SVT) with abnormal atrioventricular conduction which accounts for 15%-20% of WCT cases. The abnormal atrioventricular conduction is due to SVT with aberrant conduction, seen as a widening of the QRS complex. SVT incorporates several rhythms including inappropriate sinus tachycardia, focal and multifocal atrial tachycardia, atrial flutter, junctional tachycardia, atrio-ventircular (AV) nodal reentrant tachycardia, and various other accessory pathway-mediated reentrant tachycardias. SVT with aberrancy refers to SVT in the presence of a bundle branch block (BBB), with right BBB being more common than left BBB.

It is often difficult to distinguish ventricular tachycardia from SVT with aberrancy [6]. Many algorithms exist for distinguishing these rhythms, based on the patient’s age, cardiac history, and the morphology of the QRS complexes. However, when the rhythm diagnosis is in question, resuscitative therapy should be directed toward ventricular tachycardia, as this is the more life-threatening rhythm [6].

Electrical cardioversion can be used to treat many types of tachyarrhythmias. Table 1 summarizes the American Heart Association recommendations for energy levels for each type of arrhythmia.

**Table 1:** American Heart Association recommendations for energy levels for each type of arrhythmia VT= ventricular tachycardia, J=joules.

Arrhythmia	Energy on monophasic devices	Energy on biphasic devices
Atrial fibrillation	200 J	120-200 J
Atrial flutter	100 J	50-100 J
VT with a pulse	200 J	100 J
Ventricular fibrillation or pulseless VT	360 J	120-200 J

Cardioversion is successful in 95% of cases of ventricular tachycardia, regardless of the pathogenesis of the arrhythmia [7]. Though uncommon, cardioversion has possible complications. For example, some patients who require cardioversion have blood clots in their hearts, and electrical cardioversion has the ability to move these clots to other parts of the body [8]. Also, patients may experience additional heart rate irregularities after cardioversion [9]. Other rare complications include allergic reactions to drugs used for procedural sedation and burns or bruises from electrodes [10].

## Conclusions

Cardioversion is a life-saving procedure that can restore an irregular heart rhythm causing hemodynamic instability back to a stable cardiac rhythm. Recognizing these cardiac arrhythmias and any associated hemodynamic instability is thus of paramount importance to ensure proper blood flow to the heart.
